# Dataset of host records for introduced parasitoid wasp species (Hymenoptera) in New Zealand

**DOI:** 10.3897/BDJ.8.e59472

**Published:** 2020-11-30

**Authors:** Darren Ward, Talia Brav-Cubitt, Sarah Tassell

**Affiliations:** 1 New Zealand Arthropod Collection (NZAC) - Landcare Research, Auckland, New Zealand New Zealand Arthropod Collection (NZAC) - Landcare Research Auckland New Zealand; 2 School of Biological Sciences, University of Auckland, Auckland, New Zealand School of Biological Sciences, University of Auckland Auckland New Zealand; 3 Landcare Research, Auckland, New Zealand Landcare Research Auckland New Zealand

## Abstract

**Background:**

The introduction of species to new regions is occurring at an increasing rate. These introductions typically consist of species that are deliberately introduced for the purposes of biological control of pests or of species that are accidentally introduced through human-mediated transport networks.

Understanding the potential and actual impacts of these introduced species requires comprehensive information on their geographic distributions and biological associations.

However, apart from a few well-known case studies, such information is lacking for many introduced species which severely hinders further assessment of risks and impact.

**New information:**

A dataset is provided on host associations, geographic distributions and dates of collection for both deliberately and accidentally-introduced parasitoid wasp species (Hymenoptera) in New Zealand. Information was obtained by digitising specimens from the New Zealand Arthropod Collection. Dates of records range from 1921 to 2017.

The dataset includes 1265 specimen records, representing 127 parasitoid species from 12 families, with host records for 177 host species from 61 families and eight insect orders.

These data provide baseline information to help evaluate the risk from introduced parasitoids to non-target and native species.

## Introduction

The introduction of species to new regions is occurring at an increasing rate globally (Seebens et al. 2018). This includes species that are deliberately introduced for the purposes of biological control of pests or of species that are accidentally introduced through human-mediated transport networks.

Risk assessments are important tools used in biosecurity and pest management to provide estimates of risk towards native species (Barratt and Moeed 2005, Probert et al. 2019). However, the ability to accurately predict and quantify the risk of a species and its potential impact on native species and ecosystems is often difficult because of the lack of existing baseline data (Leung et al. 2012, Probert et al. 2019). Risk assessments are standard practice for biological control agents prior to their release (Barratt and Moeed 2005). However, risk assessments tend to disproportionately concentrate on economically-important primary sectors in comparison to native ecosystems (Probert et al. 2019). Additionally, species not involved in these economically-important agro-forestry systems typically lack basic information on their taxonomy, biology and distribution (Probert et al. 2019).

Many species of Hymenoptera are well known as invasive species, for example, ants and social wasps (Lester and Beggs 2019); whereas parasitoid Hymenoptera have received less attention despite being widely introduced (Rasplus et al. 2010; Ward and Edney-Browne 2015). A fundamental part of assessing the risk of parasitoid wasps towards native or non-target species is understanding their host associations. Typically, such information is generated by the collection and rearing of insects to see if they are parasitised. The hosts of biocontrol agents are often determined by host range testing through choice and no-choice experiments and this is a vital step of risk assessment (Schaffner 2001). A dataset, derived from records of parasitoids reared from field-collected hosts, would contribute to validating the host range hypotheses generated in the lab studies, facilitating better understanding of actual or ecological host range (Paynter and Teulon 2019). Molecular approaches also offer a new technique to inform about parasitoid-host associations (Rougerie et al. 2010; Gariepy et al. 2018).

## General description

### Purpose

The main goal was to create a comprehensive dataset of specimen records of parasitoid wasp species (Hymenoptera) introduced into New Zealand.

Taxonomic collections have accumulated specimens over long periods of time (e.g. many decades to centuries), from across many regions and landscapes and are often the only source of specific information or records of an insect species, its occurrence, distribution and specific biological associations. This is particulary so for some groups of insects, for example, cryptic and very small insects, like many parasitoid wasps, which can be overlooked in other kinds of record keeping methods, such as citizen science observations. Consequently, taxonomic collections provide key data on parasitoid-host associations based on a combination of biocontrol voucher specimens, rearing records and historical sampling.

We used species and specimens from the New Zealand Arthropod Collection (NZAC), Auckland, New Zealand. This collection is the largest in the country, has the most comprehensive coverage of the New Zealand insect fauna, being formed in 1920 (Ward 2012). Of particular relevance to this dataset are the kinds of specimens and records held in the NZAC, including: vouchers of biocontrol agents released into New Zealand; specimens and their host records from unpublished historical projects; and specimens sent for identification from members of the public, staff from government conservation and biosecurity agencies and scientists working with primary sector industries (agroforestry).

## Sampling methods

### Study extent

Specimen records from the New Zealand Arthropod Collection (NZAC), Auckland, New Zealand. This is the insect national collection of New Zealand.

### Sampling description

First, we compiled a taxonomic checklist that accounted for all species of parasitoid wasps (Hymenoptera) that have either been deliberately or accidentally introduced into New Zealand using the recent publications of Edney-Browne et al. 2017, Ward and Edney-Browne 2015. Second, all specimens of the introduced species were located in the NZAC and information on specimen labels manually transcribed into an internal collection management system to form the 'dataset'.

### Quality control

Transcription of specimen records were revised by the first author to check the status and availablity of taxonomic names. OpenRefine was used to improve data quality and standardise terms. Scientific names were checked using the New Zealand Organisms Register (NZOR).

## Geographic coverage

### Description

The dataset includes specimens only from New Zealand. The Regions of Auckland (59% of all records) and Nelson (18%) dominate the geographic coverage. This is not dissimilar to a general pattern of collecting for Hymenoptera in New Zealand (Ward 2012), but with a stronger signal from the Auckland and Nelson Regions, which is expected in this dataset as both Regions have (or have had) large research institutes related to the study of entomology, biological control and invasive insects.

### Coordinates

-49.668 and -34.692 Latitude; 178.808 and 173.009 Longitude.

## Taxonomic coverage

### Description

In total, the dataset includes specimen records of 127 parasitoid species from 12 families with host records for 177 host species from 61 families and eight insect orders. These are (alphabetically by order and family): Coleoptera (Anobiidae, Brentidae, Chrysomelidae, Coccinellidae, Curculionidae, Ptinidae); Diptera (Agromyzidae, Aleyrodidae, Calliphoridae, Cecidomyiidae, Drosophilidae, Fanniidae, Muscidae, Sarcophagidae, Sphaeroceridae, Syrphidae); Hemiptera (Aphalaridae, Aphididae, Asterolecaniidae, Cicadellidae, Coccidae, Diaspididae, Eriococcidae, Flatidae, Homotomidae, Monophlebidae, Pentatomidae, Pseudococcidae, Psyllidae); Hymenoptera (Apidae, Braconidae, Encyrtidae, Eurytomidae, Ichneumonidae, Platygastridae, Pteromalidae, Siricidae, Vespidae); Lepidoptera (Bedelliidae, Coleophoridae, Crambidae, Erebidae, Gelechiidae, Geometridae, Gracillariidae, Lymantriidae, Noctuidae, Nolidae, Nymphalidae, Oecophoridae, Pieridae, Plutellidae, Pyralidae, Roeslerstammiidae, Sesiidae, Stathmopodidae, Tineidae, Tortricidae); Neuroptera (Hemerobiidae); Orthoptera (Gryllidae); and Thysanoptera (Thripidae).

The dataset can be split into 1) 36 deliberately-introduced parasitoid species from eight families with associated records for 56 host species from 28 families; and 2) 91 accidentally-introduced parasitoid species from 11 families with host records for 138 host species from 52 families and eight insect orders.

For accidentally-introduced species, Lepidoptera has the most species as hosts (38%, Table 1), but for deliberately-introduced species, Hemiptera has the most species as hosts (40%) followed by Lepidoptera (35%). This likely reflects the importance of Hemiptera as pests and as hosts for deliberately-introduced biological control agents and possibly a sampling bias towards examining Lepidoptera as hosts of accidentally-introduced parasitoids.

## Temporal coverage

### Notes

**Figure 1.**
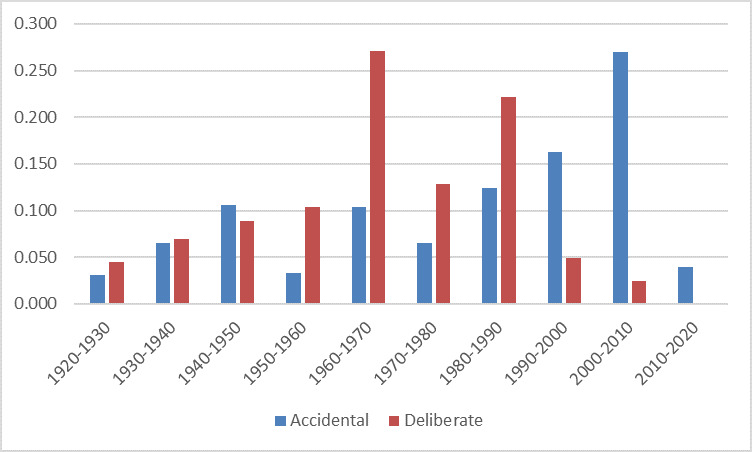
Dates of collection for specimens of accidental (blue) or deliberately (red) introduced parasitoid wasp species in New Zealand.

Overall, the temporal coverage of the dataset is from 1921 - 2017; and is similar for deliberate introductions (1922 - 2010) and accidental introductions (1921 - 2017) (Fig. 1), even though all specimens were digitised.

Most records of deliberately-introduced species are between the periods of 1960-1970s and 1980-1990s, periods that correspond with the greatest number of programmes to introduce and release biological control agents (Charles 1998).

Records of accidentally-introduced species have increased steadily over time and from 2000 onwards, dominate the dataset, reflecting the increased awareness and study of accidentally-introduced species in the general New Zealand environment (Ward and Edney-Browne 2015).

## Usage licence

### Usage licence

Creative Commons Public Domain Waiver (CC-Zero)

## Data resources

### Data package title

Introduced Parasitoid-Host Records in New Zealand

### Resource link

https://doi.org/10.7931/zsyg-1y34

### Number of data sets

1

### Data set 1.

#### Data set name

Introduced Parasitoid-Host Records in New Zealand

#### Data format

Darwin Core Standard

#### Number of columns

29

#### Download URL

https://datastore.landcareresearch.co.nz/dataset/introduced-parasitoid-host-records-in-new-zealand

#### Data format version

1.0

#### 

**Data set 1.** 

Column label	Column description
Dataset	Accidental (species unintentionally introduced into New Zealand) or Deliberate (species intentionally introduced into New Zealand, mainly for biological control)
NZAC_Accession_Number	Accession Number in the New Zealand Arthropod Collection (NZAC)
Quantity	Number of specimen records with the same information
Order	Taxonomic Order
Family	Taxonomic Family
Taxon_Name	Genus and species name
Life_Stage	Life stage of digitised specimens (all are Adults)
Country	Country from where specimen was collected (all records are from New Zealand)
New_Zealand_Area_Code	2-digit area code of entomological regions in New Zealand
Locality	Location the specimen was collected from
Date	Date the specimen was collected
Year	Year the specimen was collected
Collectors	Person(s) who collected the specimen(s)
Method	How the specimen was collected / sampled
Biological_information_label	Biological information on the specimen label
Has_Host	Yes /No
Host_species	Genus and species name of the host species
Host_NZ_Biostatus	Status of host in NZ: Endemic (only found in NZ); Native (naturally occurring in NZ, but also present elsewhere) or Exotic (accidentally or deliberately introduced into New Zealand)
Host_Common_Name	Name on specimen label when a taxonomic name was not listed
Identification_Uncertainty	Uncertainty of the host identification is noted
Host_Family	Taxonomic Family of the host
Host_Order	Taxonomic Order of the host
Life_Stage_of_Host	Life Stage of Host recorded on specimen label (adult, larvae, pupa, immature)
Association	Other information on specimen label relating to host/biology/microhabitat
Latitude_Degrees_decimal	Decimal degrees of latitude
Longitude_Degrees_decimal	Decimal degrees of longitude
Datum	Georeference system (all are WGS84)
Is_derived	Is the georeferenced derived
Measurement_Method_Name	Method to obtain derived georeference

## Figures and Tables

**Table 1.** Percentage of host records for each taxonomic order for accidentally and deliberately-introduced parasitoid species in New Zealand.

Order	Accidental Introductions	Deliberate Introductions
Coleoptera	13%	7%
Diptera	16%	16%
Hemiptera	20%	40%
Hymenoptera	13%	2%
Lepidoptera	38%	35%
Orthoptera	1%	0%
Thysanoptera	1%	0%
